# Conotoxin Gene Superfamilies

**DOI:** 10.3390/md12126058

**Published:** 2014-12-17

**Authors:** Samuel D. Robinson, Raymond S. Norton

**Affiliations:** Medicinal Chemistry, Monash Institute of Pharmaceutical Sciences, Monash University, Parkville, Melbourne, VIC 3052, Australia; E-Mail: ray.norton@monash.edu

**Keywords:** conotoxin, gene superfamily, conopeptide, *Conus*, venom, toxin

## Abstract

Conotoxins are the peptidic components of the venoms of marine cone snails (genus *Conus*). They are remarkably diverse in terms of structure and function. Unique potency and selectivity profiles for a range of neuronal targets have made several conotoxins valuable as research tools, drug leads and even therapeutics, and has resulted in a concerted and increasing drive to identify and characterise new conotoxins. Conotoxins are translated from mRNA as peptide precursors, and cDNA sequencing is now the primary method for identification of new conotoxin sequences. As a result, gene superfamily, a classification based on precursor signal peptide identity, has become the most convenient method of conotoxin classification. Here we review each of the described conotoxin gene superfamilies, with a focus on the structural and functional diversity present in each. This review is intended to serve as a practical guide to conotoxin superfamilies and to facilitate interpretation of the increasing number of conotoxin precursor sequences being identified by targeted-cDNA sequencing and more recently high-throughput transcriptome sequencing.

## 1. Introduction

Conotoxins (or conopeptides) are the peptidic components of the venoms of marine cone snails (genus *Conus*). These venoms are remarkably complex, each containing more than 100 unique conotoxins. These peptides have attracted enormous interest from biomedical researchers as many of those characterised display unprecedented potency and selectivity for their molecular target, which can include ion channels, G protein-coupled receptors (GPCRs) or neurotransmitter transporters [1,2]. Conotoxins have thus proven to be valuable as research tools as well as drug leads and therapeutics. As a consequence, there has been a concerted and increasing drive for the identification and characterisation of new conotoxins. Currently, these peptides are classified in one of three ways: gene superfamily, cysteine framework or pharmacological family. Historically, conotoxins were also divided into those that were “cysteine-rich” and those that were “cysteine-poor”, the latter being termed conopeptides, but this distinction is now considered redundant [3].

In many ways the current classifications reflect how conotoxins have been identified. Much of the early research effort into conotoxins focused on their isolation directly from venom, usually by assay-directed fractionation. Accordingly, pharmacological family and cysteine framework were the primary methods of conotoxin classification. The pharmacological family classification was based on the receptor target and type of interaction of a conotoxin. The cysteine framework classification refers to a characteristic arrangement of cysteine residues in a conotoxin’s primary structure (independent of their connectivity), and, to date, 26 distinct cysteine frameworks have been described in conotoxins.

**Figure 1:**
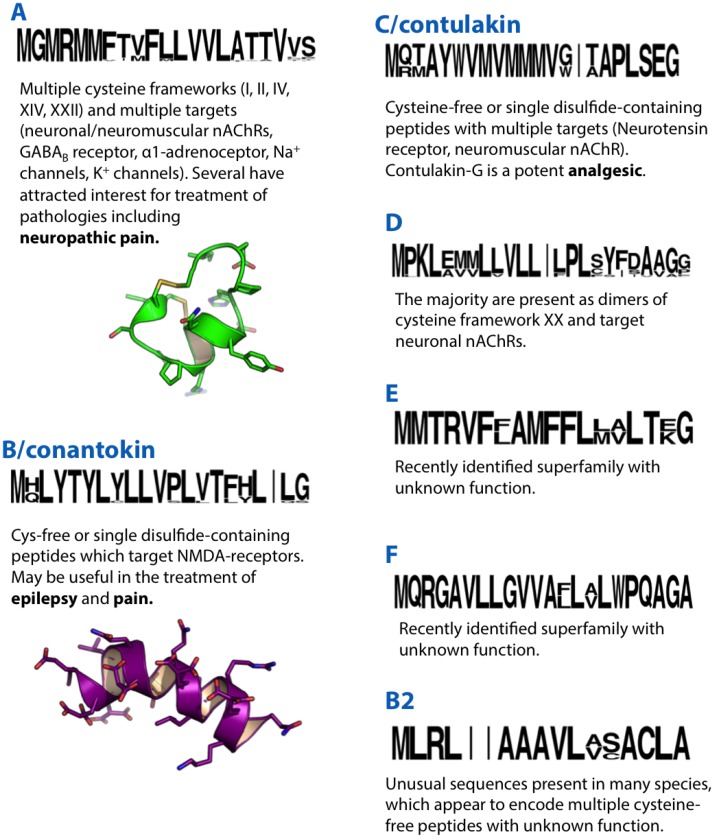
Summaries of the A, B, B2, C, D, E and F conotoxin superfamilies. Sequence logos illustrate the precursor signal peptide sequence that defines each superfamily. Sequence logos in this and subsequent figures were generated based on available precursor sequences from Conoserver [11] and/or Uniprot/Genbank. Structures of the A-superfamily conotoxin Vc1.1 (PDB ID: 28HS) and B-superfamily conantokin-G (10NU) are shown in green and purple, respectively.

**Figure 2:**
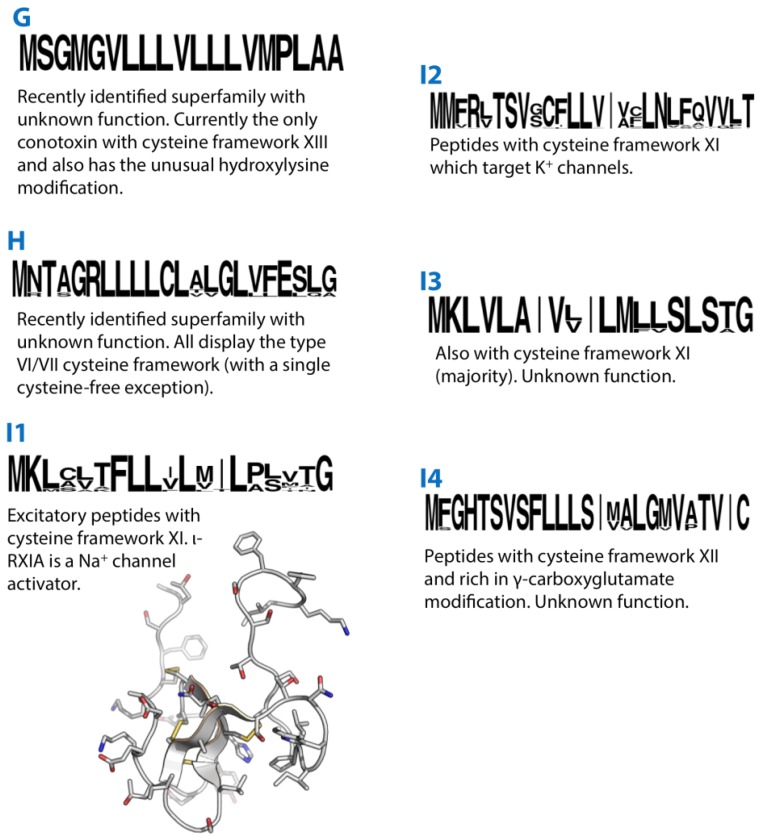
Summaries of the G, H, I1, I2, I3 and I4 conotoxin superfamilies. Sequence logos illustrate the precursor signal peptide sequence that defines each superfamily. The structures of the I1-superfamily conotoxin ι-RXIA (2JRY) is shown in grey.

**Figure 3:**
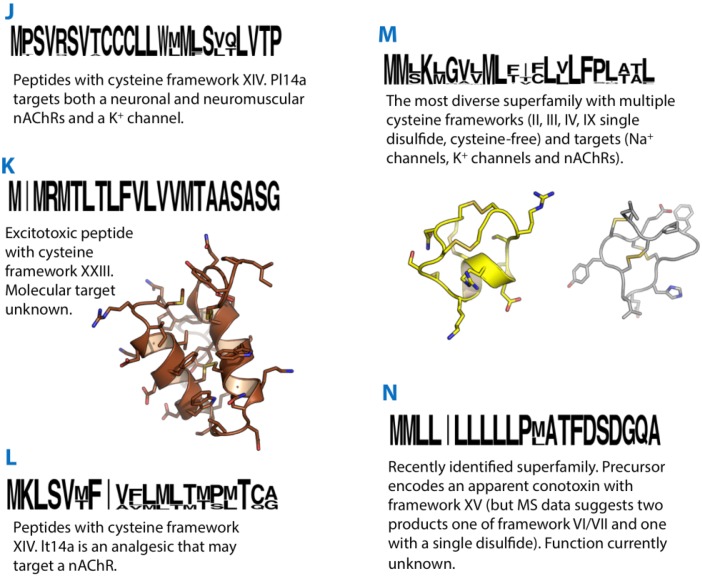
Summaries of the J, K, L, M and N conotoxin superfamilies. Sequence logos illustrate the precursor signal peptide sequence that defines each superfamily. Structures of the K-superfamily conotoxin Im23a (2LMZ) and M-superfamily conotoxins KIIIA (2LXG) and mr3e (2EZF) are shown in brown, yellow and grey, respectively.

**Figure 4:**
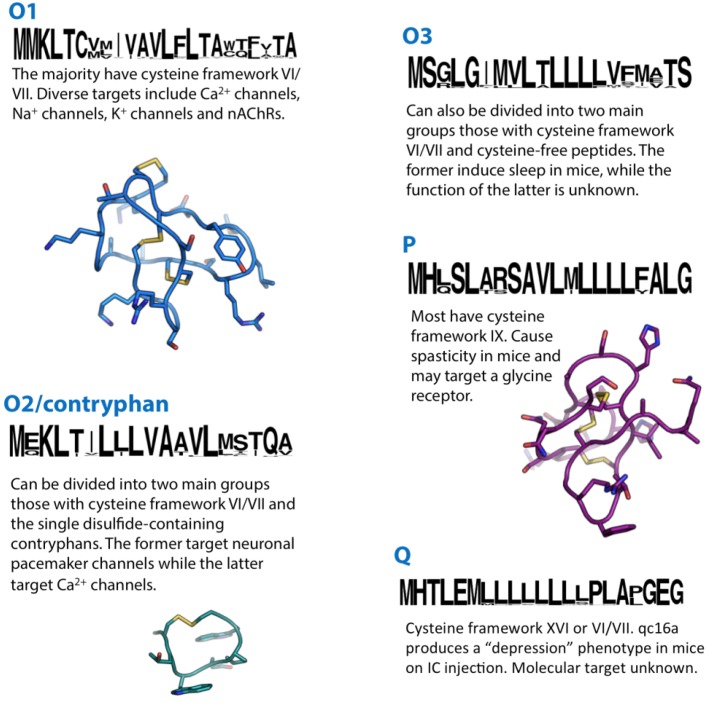
Summaries of the O1, O2, O3, P and Q conotoxin superfamilies. Sequence logos illustrate the precursor signal peptide sequence that defines each superfamily. Structures of the O1-superfamily conotoxin ω-MVIIA (1TTK), the O2-superfamily contryphan-R (1QFB) and the P-superfamily conotoxin gm9a (1IXT) are shown in blue, cyan and purple, respectively.

**Figure 5:**
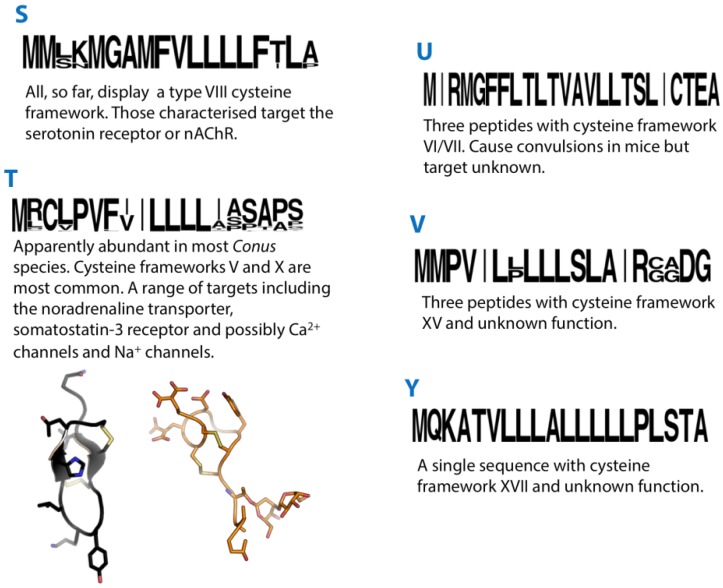
Summaries of the S, T, U, V and Y conotoxin superfamilies. Sequence logos illustrate the precursor signal peptide sequence that defines each superfamily. Structures of the T-superfamily conotoxins χ-MrIA (2EW4) and TxVA (1WCT) are shown in black and orange, respectively.

**Figure 6:**
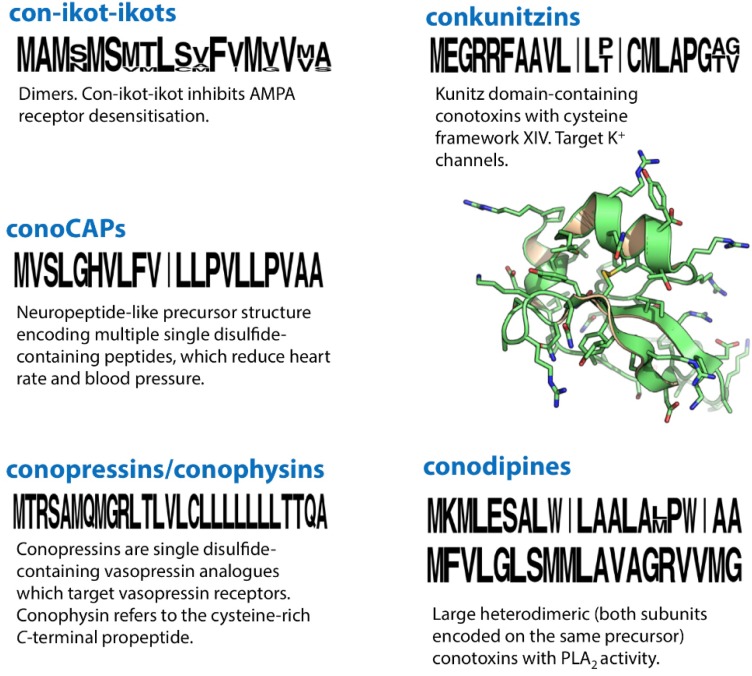
Summaries of the con-ikot-ikot, conoCAP, conopressin, conkunitzin and conodipine conotoxin superfamilies. Sequence logos illustrate the precursor signal peptide sequence that defines each superfamily. The structure of conkunitzin-S1 (1Y62) is shown in green.

Conotoxins are translated from mRNA as peptide precursors, typically with a characteristic three-domain organization consisting of an *N*-terminal signal peptide, followed by a propeptide then a single copy of the mature peptide encoded near the *C*-terminus. The signal peptide sequence, which constitutes the *N*-terminus of precursor peptides, is characterised by a series of about 20 hydrophobic amino acids, often including one or more positively-charged residues, and is responsible for targeting the precursor peptide to the cellular secretory pathway. The propeptide is thought to play a role in conotoxin maturation, and is removed prior to secretion of the mature conotoxin peptide. Following the first sequencing of full conotoxin precursors [4], it was recognized that different conotoxins were related based on high sequence similarity in their signal and propeptide regions. This led to the grouping of conotoxins into “gene superfamilies” based on this consensus signal peptide sequence. Other than the expected general features of signal peptides, there is little sequence similarity between the different superfamilies. The signal peptide sequences of each of the described conotoxin superfamilies are presented in Figure 1, Figure 2, Figure 3, Figure 4, Figure 5 and Figure 6. cDNA sequencing is now the primary method for identification of new conotoxin sequences and, as a result, gene superfamily has become the most convenient method of conotoxin classification.

By definition, conotoxins within a superfamily share a similar signal peptide sequence, but, as this review highlights, there is remarkable structural and functional diversity in the encoded mature peptides. Gene duplication followed by diversifying selection has been proposed as a mechanism responsible, at least in part, for the observed hypervariability in mature peptide sequences within a conotoxin superfamily [5,6,7]. Accordingly, where multiple sequences are reported for a given superfamily, a proportion might be expected to represent non-functional pseudogenes. In practice, however, this is difficult to verify, as most conotoxins are not assayed against an exhaustive array of possible targets. For many uncharacterised conotoxins, superficial similarity to known conotoxins, high transcript expression levels and their presence in the venom are strongly suggestive of bioactivity.

This review is structured as a collection of short summaries of each of the conotoxin gene superfamilies described so far, as well as several conotoxins yet to be classified. Each section highlights the structural and functional diversity of each superfamily and the research performed to uncover this information. Relatively obscure conotoxin groups are intentionally covered in more detail, while the more thoroughly-studied conotoxin groups (e.g., the A-superfamily α-conotoxins), which are already the subject of recent comprehensive reviews [8,9,10], are not covered in detail and the reader is directed to those reviews.

This review has been designed to act as a resource to serve those researching conotoxins. Its main purpose is to facilitate interpretation of the vast and increasing number of conotoxin sequences being identified by targeted-cDNA sequencing and, more recently, high-throughput transcriptome sequencing. For example, researchers faced with novel conotoxin precursor sequences can use the information provided herein to quickly and easily identify whether these are members of known superfamilies or represent new superfamilies. If the sequences are members of known superfamilies, what is already known about them, what are the key citations, and can assumptions on their likely function be justified?

The general trends in structure, function, and diversity of known conotoxins have been reviewed recently [11]. Similarly, an analysis of all available conotoxin signal sequences using a phylogenetic approach was recently reported [3]. This work is intended to complement these efforts and to serve as a practical guide to conotoxin gene superfamilies. We also note that the collation of these data would have been substantially more difficult without the outstanding efforts already made by those in setting up and maintaining the conotoxin database Conoserver [11].

## 2. A-Superfamily

α-GI, a peptide of 13 amino acids with two disulphide bonds was among the first conotoxins to be isolated from *Conus* venom [12]. Further characterisation revealed that this peptide was a nicotinic-acetylcholine receptor (nAChR) antagonist, inhibiting neuromuscular transmission [13]. Several years later it was recognised that α-GI was part of a diverse group of conotoxins sharing a similar signal peptide sequence which were subsequently designated the A-superfamily [14]. Like α-GI, the majority of A-superfamily conotoxins are characterised by the type I cysteine framework (CC-C-C) and are known to potently and selectively target an array of neuronal and neuromuscular nAChR subtypes. This group of conotoxins, which target nAChRs, was named α-conotoxins, and many have now been characterised in detail, proving valuable as pharmacological probes of nAChR function. As they are already the subject of an excellent review [8], the remainder of this section will focus on non-canonical A-superfamily conotoxins.

The full precursor sequence of α-GI was revealed using a degenerate primer based on the mature amino acid sequence [14]. Primers were then designed based on the 5′ and 3′ untranslated regions (UTRs) of this sequence and targeted cDNA sequencing was performed on venom duct cDNA libraries of several other species in an effort to identify other α-conotoxins. This approach confirmed the presence, in the venoms of other species, of other A-superfamily conotoxins. Surprisingly, however, several sequences displayed the same A-superfamily signal peptide sequence, but encoded longer predicted mature peptides of type IV cysteine framework (C-C-C-C-C-C) (SIVA, SIVB, MIVA, SmIVA and SmIVB) with virtually no similarity to the α-conotoxins. These conotoxins differed not only in primary structure but also in their function. Of these, SIVA had been previously isolated from *Conus striatus* venom [15], and, in contrast to the α-conotoxins, was a K^+^ channel blocker. It also displayed a remarkable array of post-translational modifications, including a pyroglutamylated *N*-terminus, an amidated *C*-terminus, three hydroxyprolines and a glycosylated serine.

While it was established that SIVA was a K^+^ channel blocker, other conotoxins similar in primary structure to SIVA displayed different functions. CcTx, a conotoxin isolated from the venom of *Conus consors*, with a type IV cysteine framework and similar primary structure to SIVA, including several post-translational modifications, was shown to target neuronal voltage-gated Na^+^ channels (VGSCs) [16]. The first framework IV conotoxin to be isolated, PIVA [17], which was only later found to be a member of the A-superfamily [18], was an nAChR antagonist. Similarly, OIVA and OIVB, purified from the venom of *Conus obscurus*, are antagonists of neuromuscular nAChRs [19]. Interestingly, these conotoxins were shown to be selective for the foetal subtype of neuromuscular nAChR, making them potentially important tools for delineating the roles of these receptors [18,20].

Two conotoxins isolated recently from the venom of *Conus purpurascens*, PIVE and PIVF, displayed excitatory activity upon injection into fish but displayed no detectable activity when injected into mouse brain or at various K^+^ channel subtypes in *Xenopus* oocytes [21]. It should be noted that their assignment as A-superfamily conotoxins was based not on experimental evidence but rather on similarity in primary structure and cysteine framework to those already identified.

The α-conotoxin SII, an inhibitor of neuromuscular nAChRs isolated from the venom of *C. striatus*, displayed the unusual type II cysteine framework (CCC-C-C-C) [22]. cDNA sequencing subsequently revealed that this conotoxin was a member of the A-superfamily [23]. SII remains the only A-superfamily conotoxin identified with a type II cysteine framework. One other conotoxin with this framework has been identified in the M-superfamily (described below). The disulphide connectivity and three-dimensional structure of framework II conotoxins are yet to be established.

The A-superfamily conotoxin Pu14.1, identified by cDNA sequencing [24], displayed a type XIV cysteine framework (C-C-C-C) in its predicted mature peptide. The disulphide connectivities of the synthesised and folded predicted mature peptide were determined as I-III, II-IV. The synthetic peptide induced unconsciousness in mice (intravenous injection) and seizures, paralysis and death in fish upon intramuscular (IM) injection. Electrophysiology assays showed the peptide was an α-conotoxin inhibiting neuronal and neuromuscular nAChRs. A similar conotoxin ts14a isolated from the venom of *Conus tessulatus* also displayed a 1-3, 2-4 connectivity (deduced by mass spectrometry following CNBr cleavage) and is most likely an A-superfamily conotoxin related to Pu14.1.

Molecular targets of A-superfamily conotoxins are not limited to ion channels. A single A-superfamily conotoxin (ρ-TIA) was shown to target the α1-adrenoceptor, a GPCR [25]. This peptide shares the cysteine framework, disulphide connectivity and overall fold of the A-superfamily α-conotoxins, but differs markedly in the amino acid composition of its inter-cysteine loops. Similarly, a subset of framework I A-superfamily conotoxins including Vc1.1, RgIA, PeIA and AuIB, not only antagonise nAChRs, but also indirectly inhibit N-type voltage-gated Ca^2+^ channel (VGCC) function by acting as agonists of the γ-aminobutyric acid (GABA)_B_ GPCR. These have been reviewed recently [26].

A single A-superfamily precursor identified in the venom gland transcriptome of *Conus victoriae* (A_Vc22.1) encodes a predicted mature peptide that exhibits a remarkably different primary structure to known A-superfamily conotoxins, with eight cysteines arranged in a type XXII cysteine framework (C-C-C-C-C-C-C-C) [27]. Similarly, a single A-superfamily precursor identified from *Conus flavidus* encoded a predicted mature peptide with cysteine framework VI/VII (C-C-CC-C-C) [28]. A summary of pharmacological activities associated with selected A-superfamily conotoxins is presented in Table 1.

**Table 1:** Activities of selected A-superfamily conotoxins.

	Sequence	Activity	Reference
GIA	E**CC**NPA**C**GRHYS**C**GK	inhibits neuromuscular nAChR	[12]
Vc1.1	G**CC**SDPR**C**NYDHPEI**C** *	inhibits neuronal nAChR (α9α10) and GABA_B_ GPCR	[29]
TIA	FNWR**CC**LIPA**C**RRNHKKF**C** *	α1-adrenoceptor modulator	[25]
SII	G**CCC**NPA**C**GPNYG**C**GTS**C**S	inhibits neuromuscular nAChR	[22]
PIVA	G**CC**GSYONAA**C**HO**C**S**C**KDROSY**C**GQ *	inhibits neuromuscular nAChR	[17]
SIVA	ZKSLVPSVITT**CC**GYDOGTM**C**OO**C**R**C**TNS**C** *	K^+^ channel blocker	[15]
CcTx ^#^	AOWLVPSQITT**CC**GYNOGTM**C**OS**C**M**C**TNT**C**	activates neuronal VGSCs	[16]
EIVA	G**CC**GPYONAA**C**HO**C**G**C**KVGROOY**C**DROSGG *	inhibits foetal and adult neuromuscular nAChRs	[18]
OIVA	**CC**GVONAA**C**HO**C**V**C**KNT**C** *	selectively inhibits foetal neuromuscular nAChRs	[18]
OIVB	**CC**GVONAA**C**PO**C**V**C**NKT**C**G *	selectively inhibits foetal neuromuscular nAChR	[18]
PIVE ^#^	D**CC**GVKLEM**C**HP**C**L**C**DNS**C**KNYGK *	excitatory activity upon injection into fish	[21]
Pu14.1	VLEKD**C**PPHPVPGMHK**C**V**C**LKT**C**	inhibits neuronal and neuromuscular nAChRs	[24]

Z, pyroglutamic acid; S, glycosylated serine; O, hydroxyproline; *, *C*-terminal amidation. ^#^, Tentative assignment to the A-superfamily was based on similarity in primary structure and cysteine framework.

## 3. B/Conantokin-Superfamily

The sleeper peptide (or conantokin-G) was isolated from the venom of *Conus geographus* based on its ability to induce a “sleeping” phenotype on intracranial (IC) injection in mice [30]. Conantokin-G was the first conotoxin identified that did not have cysteine residues and was unusual in that it contained five γ-carboxyglutamate modifications, which was also the first time this modification was observed in conotoxins [31]. It was later shown that these γ-carboxyglutamate modifications induced α-helicity of the linear peptide in the presence of divalent cations [32].

Importantly, a developmental switch in the reaction to this peptide was observed, such that mice younger than two weeks displayed the “sleeping” phenotype while those older than three weeks displayed a hyperactive phenotype [33]. It was noted that the hyperactive phenotype induced by conantokin-G in older mice resembled the behavioural effects induced by non-competitive *N*-methyl-D-aspartate receptor (NMDAR) antagonists [34]. Thus, conantokin-G was examined for activity and shown to be a potent and selective NMDAR antagonist but with a mechanism distinct from those already described. Conventional NMDARs are tetrameric, generally composed of two NR1 subunits and two of either of the four subtypes of NR2 (A-D) [35]. NR3 subunits also exist and can modulate conventional NR1/NR2 NMDAR activity or combine with the NR1 subunit to form a type of excitatory glycine receptor [36]. In conventional NMDARs it is the NR2 component that is regulated developmentally and spatially and is responsible for the pharmacological properties of the receptor. Conantokin-G was shown to be selective for NMDARs containing the NR2B subunit [37].

Several other conantokin peptides have since been identified and characterised and all display NMDAR antagonism with preferential selectivity for NR2B-containing NMDARs. They do differ, however, in their degree of selectivity for the other three NR2 subtypes [38,39,40,41]. There is potential for the development of selective NMDAR ligands based on conantokins. Such ligands are in demand as research tools for delineating the role of individual NMDAR subtypes in the central nervous system.

Conantokins have also attracted considerable interest as analgesics, anticonvulsants and for the treatment of other neurological disorders [42]. Indeed, conantokin-G reached phase I clinical trials for the treatment of both pain and epilepsy [43].

It has been proposed that conantokins with activity at human NMDARs are unique to piscivorous *Conus* species [44]. Although several conantokins have been identified in both vermivorous and molluscivorous species [27,28] these peptides are generally more diverse in amino acid composition than conantokins from piscivorous species, and to date there are no published data on their molecular target.

## 4. B2-Superfamily

The first member of this superfamily (Uniprot Q2HZ30) was identified as a highly expressed sequence in a *Conus litteratus* venom gland cDNA library and termed high frequency protein-1 [45]. A clearly related sequence was subsequently identified in the transcriptome of *C. consors* and matched to several linear peptides in the venom [46]. Confirmation that this group of high-frequency peptides was widespread in *Conus* came with the recent identification of several similar sequences in the venom gland transcriptomes of three other species—*C. geographus*, *C. victoriae* and *Conus bullatus* [27]. Although bioactivity of the peptide products of these unusual sequences has not been demonstrated, they have been assigned in Conoserver [11] as a new “B2-superfamily”.

## 5. B3-Superfamily

A sequence, termed αB-VxXXIVA, was recently reported from a *Conus vexillum* venom gland cDNA library [47]. A mature peptide from the putative precursor sequence was conjectured and synthesised with each of the three possible disulphide arrangements (excluding the possibility of dimerisation). Two of the synthetic peptides displayed μM affinity for the α9α10 subtype of nAChR, while all of them displayed largely disordered structures in aqueous solution. Despite the lack of a clear signal peptide sequence this single precursor has been designated a new “B3-superfamily”.

## 6. C/Contulakin-Superfamily

Isolated from *C. geographus*, the 16 residue contulakin-G (CGX-1160) was the first member of the neurotensin family to be identified from an invertebrate source [48]. Neurotensin is an analgesic that activates neurotensin GPCRs, which play an important role in neurotransmission and neuromodulation. Contulakin-G induced sluggish behaviour upon IC injection into mice, and was subsequently found to bind to the human neurotensin type 1 receptor, rat neurotensin type 1 and 2 receptors and the mouse neurotensin type 3 receptor (Table 2). Contulakin-G has no disulphide bonds but has two post-translational modifications, pyroglutamate and *O*-glycosylation of Thr, the latter of which had not previously been observed in conotoxins. The *O*-glycosylation is important for the peptide’s biological activity. Contulakin-G elicited analgesia in two preclinical models of nociception and displayed no unfavourable cardiovascular or motor effects [49], suggesting its potential as an intrathecally delivered analgesic and supporting its progression to phase I clinical trials.

A 32-amino acid conotoxin with a single disulphide bond, αC-PrXA, isolated from the venom of *Conus parius*, also displays the C-superfamily signal peptide sequence [50]. In contrast to contulakin-G, αC-PrXA is a highly specific inhibitor of neuromuscular nAChRs.

Since the initial discovery of contulakin-G, many precursor sequences have been identified from a variety of *Conus* species [51]. The conotoxins derived from these precursors were termed β-conotoxins and, while the majority displayed single disulphides, others displayed cysteine frameworks XIV and V (CC-CC). Furthermore, they were reportedly potentially useful for the treatment of disorders involving voltage-gated ion channels, ligand-gated ion channels and/or receptors, but there are no published data on their activity. Two more C/contulakin precursor sequences have been discovered in *C. litteratus*, contulakin-Lt1 and contulakin-Lt2 [45], and one in *Conus pulicarius* [52].

**Table 2:** Activity of C-superfamily conotoxins.

	Sequence	Activity	Reference
Contulakin-G	ZSEEGGSNATKKPYIL	targets neurotensin receptor	[48]
αC-PrXA	TYGIYDAKPOFS**C**AGLRGG**C**VLPONLROKFKE *	inhibitor of neuromuscular nAChRs	[50]

Z, pyroglutamic acid; T, glycosylated threonine; O, hydroxyproline; *, *C*-terminal amidation.

## 7. D-Superfamily

VxXXA, VxXXB and VxXXC were initially isolated from the venom of *C. vexillum* as ~11 kDa components that inhibited nAChRs [53]. These unusually large native conotoxins occur as pseudo-homodimers of paired 47–50 residues. Binding assays and two-electrode voltage clamp analyses indicated that they were subtype-selective inhibitors of α7 and β2-containing nAChRs. Subsequent cloning of several D-superfamily conotoxin precursor sequences indicated that they indeed constituted a new “D-superfamily” [54], one that is so far limited to several vermivorous species of *Conus*. Several D-superfamily precursor sequences with a type XV cysteine framework (C-C-CC-C-C-C-C) were also recently reported from *C. litteratus*.

## 8. E-Superfamily

The E-superfamily of conotoxins was recently discovered in the venom gland transcriptomes of *Conus marmoreus* [55] and *C. victoriae* [27]. This superfamily, at present, consists of a single sequence from each species. The peptide product of Mr104 from the venom of *C. marmoreus*, is 26 amino acids in length, with four cysteines (two disulphide bonds) and a bromotryptophan. No function has yet been identified for peptides derived from E-superfamily precursors, although superficial similarity to other conotoxins, high expression levels and their presence in the venom are consistent with a role as toxins in envenomation.

## 9. F-Superfamily

The F-superfamily of conotoxins, like the E-superfamily, was recently discovered in the venom gland transcriptomes of *C. marmoreus* [55] and *C. victoriae* [27] and again is defined by only a single sequence from each species. A peptide product was identified for the F-superfamily precursor in *C. marmoreus* (Mr105), although this short linear peptide was derived from the putative propeptide sequence. As with the E-superfamily, no function has yet been identified for peptides derived from F-superfamily precursors.

## 10. G-Superfamily

The peptide de13a isolated from the venom of *Conus delessertii* is the only conotoxin so far identified that displays a type XIII cysteine framework (C-C-C-CC-C-C-C) [56]. It was also unique in that it contained a hydroxyl-lysine modification. Using the sequence information of the isolated de13a, a precursor sequence (De13.1) was recently identified from *C. delessertii* venom gland cDNA [57]. This revealed a unique signal peptide sequence (although with some similarity to that of the O3-superfamily) and subsequently de13a and De13.1 were assigned to a new “G-superfamily”. *C. delessertii* is one of several *Conus* species recently classified into a separate Conasprella clade [58] and it remains to be established whether the G-superfamily is found in other *Conus*.

## 11. H-Superfamily

The H-superfamily is a recently described group of conotoxins from *C. marmoreus* [55] and *C. victoriae* [27]. The majority of H-superfamily conotoxins identified so far share the type VI/VII cysteine framework common to the O1, O2 and O3 (and U) superfamilies. A single sequence in *C. victoriae* encodes a cysteine-free peptide product.

## 12. I1-Superfamily

I1-superfamily conotoxins are relatively large and display a type XI cysteine pattern (C-C-CC-CC-C-C). I1 conotoxins tested to date exhibit excitatory activity [59]. Of these, ι-RXIA, a 46-amino acid peptide, is the best characterised, and is an activator of Na_V_1.6 and Na_V_1.2 VGSCs [60,61]. Structurally, the peptide has an inhibitor cysteine knot (ICK) motif with an additional disulphide. Several I1 conotoxins, including RXIA, contain a D-amino acid modification that is functionally important [62].

## 13. I2-Superfamily

I2-superfamily conotoxins share the same cysteine pattern as the I1-superfamily but are K^+^ channel modulators. ViTx and sr11a are selective inhibitors of K_V_1.1 and 1.3 [63] and K_V_1.2 and 1.6 channels [64], respectively, while BeTX is a potentiator of the Ca^2+^- and voltage-dependent BK channel [65]. I2-superfamily conotoxins have an unusual precursor structure in that the encoded mature peptide directly follows the signal peptide, while a propeptide region is found at the *C*-terminus of the precursor.

## 14. I3-Superfamily

A recently described third I-superfamily (I3) shares the cysteine framework of the I1 and I2-superfamilies but displays a distinct signal peptide sequence [66]. Two peptides, ca11a and ca11b, were initially isolated from the venom of *Conus caracteristicus*. These did not display any post-translational modifications. This sequence information was used to sequence cDNA encoding other I3-superfamily conotoxins from two other vermivorous species, two of which displayed the type VI/VII cysteine framework most commonly associated with the O1, O2 and O3 superfamilies.

## 15. I4-Superfamily

The I4-superfamily of conotoxins was previously grouped with the I2-superfamily, despite the fact that these conotoxins have a clearly distinct signal peptide sequence and also exhibit a distinct cysteine pattern (framework XII, C-C-C-C-CC-C-C) compared to I2-superfamily conotoxins [67]. This disparity was noted previously [68], where the authors proposed that this group of peptides be redefined as “E-conotoxins”. Given the superficial similarity of these conotoxins to other I-superfamilies they have now been designated as the “I4-superfamily” [27].

I4-superfamily conotoxin precursors are unusual in that like the I2-suerfamily the mature peptide is located between the signal and propeptide regions. The mature peptides are rich in γ-carboxyglutamate modifications (five each in Gla-MrII and Gla-TxX) [67]. The activity of I4-superfamily conotoxins remains to be determined.

## 16. J-Superfamily

The isolation and characterisation of conotoxin pl14a from the cone snail *Conus planorbis* marked the discovery of the J-superfamily [69]. The conotoxin contains 25 amino acid residues with an amidated *C*–terminus and an elongated *N*-terminal tail. The confirmed cysteine pattern was C-C-C-C (type XIV) with a I-III, II-IV disulphide connectivity, a novel framework distinct from any previously characterised conotoxins. Solution structure determination by NMR spectroscopy revealed a highly helical structure that was unique among conotoxins. The cDNA clone encoding the precursor had a unique signal sequence, indicating that pl14a belonged to a new gene superfamily, which was named the J-superfamily. From this signal sequence, a further five J-conotoxins were identified from *C. planorbis* and *Conus ferrugineus* with similarities in predicted peptide length, loop size and *C*-terminal amidation.

The J-superfamily conotoxin pl14a caused shaking, barrel-rolling, seizures and at higher doses death in mice following IC injection. It displayed potent inhibitory effects at both nAChR subtypes (α3β4-neuronal, α1β1εδ-neuromuscular) and a voltage-gated potassium channel subtype (K_V_1.6). Interestingly, it was the first conotoxin to have shown activity at both voltage-gated and ligand-gated ion channels. Since the discovery of pl14a, several other J-superfamily precursor sequences have been identified in other *Conus* species [27,70].

## 17. K-Superfamily

Two conotoxins (im23a and im23b) isolated from the venom of *Conus imperialis* marked the discovery of not only the K-superfamily, but also a new cysteine framework XXIII (-C-C-C-CC-C-) [71]. Im23a was expressed recombinantly and found to adopt a helical hairpin fold with a cluster of acidic residues on the surface that may play a role in calcium binding. The disulphide connectivity was determined as I-II, III-IV, V-VI by chemical mapping and NMR structure calculations. Targeted cDNA sequencing for K-superfamily sequences in other *Conus* species was performed, resulting in one sequence each from *Conus quercinus*, *Conus virgo* and *C. marmoreus*, all with high similarity to im23a and im23b. Mice injected IC with either im23a or im23b displayed excitatory symptoms, but the molecular target of these conotoxins remains unknown.

## 18. L-Superfamily

The defining member of the L-superfamily, lt14a, was identified in a cDNA library of *C. litteratus* [72]. It displayed a unique signal sequence and the cysteine framework XIV. Based on the predicted mature peptide sequence of lt14a, a peptide was synthesised with the globular disulphide arrangement. This synthetic peptide inhibited an undetermined subtype of nAChR and displayed analgesic activity in a mouse hot-plate test. L-superfamily sequences have since been identified in the piscivorous *Conus eberneus* [70] and *C. geographus* [73] (where a single L-superfamily sequence (G61) is referred to as the Z-superfamily), the vermivorous *C. pulicarius* [52] as well as in *Conus miles* where the corresponding 11-residue mature peptide was confirmed by MS/MS matching [74].

Several conotoxin sequences from *Conus californicus* [75], currently listed in Conoserver [11] as members of the L-superfamily appear to differ in their signal peptide sequence from previously defined L-superfamily conotoxins, although they share the same cysteine framework.

## 19. M/Conomarphin/CPY-Superfamily

The M-superfamily of conotoxins is the subject of a recent review [9], so will only be covered briefly here, with an emphasis on recent developments. The majority of M-superfamily peptides identified display a type III cysteine pattern, CC-C-C-CC, while conomarphins and conopeptide-Y are cysteine-free. The M-superfamily has been further subdivided into the M1, M2, M3, M4, and M5 groups, according to the number of residues present in the third intercysteine loop. Although all share the same basic pattern, the M1 and 3 subgroups share a more similar precursor sequence, as do the M2, M4 and M5 subgroups, while the conomarphins are slightly different again.

M-superfamily conotoxins for which a molecular target has been identified are largely limited to the M4 and M5 branches. These include µ-conotoxins (which block VGSCs), κM-conotoxins (which block voltage-gated K^+^ channels) and ψ-conotoxins (which block nAChRs). µM-conotoxins that selectively target neuronal-type VGSCs show considerable potential as drug leads in the development of new analgesics [76,77]. The two κM-conotoxins, RIIIJ and RIIIK, both isolated from the venom of *Conus radiatus* [78,79,80], selectively block the K_V_1.2 subtype of K^+^ channels (RIIIJ with 10-fold higher potency) and are potentially valuable tools for understanding mechanisms of cardioprotection. The three ψ-conotoxins (PIIIE, PIIIF and PrIIIE) are nAChR antagonists but bind at a distinct site from that of the α-conotoxins [81,82,83] and may prove useful tools for the characterisation of a new target for drug development.

While the M4/5 branch of conotoxins is well characterised, there are limited published data describing the M1 and M2 branches. Much of what is known of the M1 branch comes from studies performed on two peptides mr3e [84,85] and tx3a [86]. mr3e, from the molluscivorous *C. marmoreus* [84], consists of 16 residues and has a disulphide connectivity of I-V, II-IV, III-VI, different from most M2 and M4 conotoxins (with the recently reported exception of KIIIA [87]). tx3a, a 15-residue peptide identified in the molluscivorous *Conus textile*, shares the same disulphide connectivity as mr3e. Structurally, mr3e can be described as a “flying bird motif” [88] while tx3a forms a very different “triple-turn motif” [86]. While, mr3e has no obvious effects on IC injection in mice, tx3a causes excitatory behaviour, producing hyperactivity at low doses and seizures and death at higher doses [86]. Recently, an M1 conotoxin isolated from the venom of *C. litteratus*, was shown to enhance TTX-sensitive Na^+^ currents in a whole-cell patch clamp assay [89], perhaps indicating the molecular target of this group of conotoxins. All of the M2 conotoxins tested so far (tx3b and c, and mr3a and b) elicited excitatory symptoms upon IC injection in mice [90], although a molecular target is yet to be identified. The three-dimensional structure of mr3a is described as containing a “triple-turn” motif [85] with a disulphide connectivity of I-VI, II-IV, III-V [90]—different to M1 and M4 conotoxins. Recently, the three-dimensional structure of another M2 conotoxin, BtIIIA, was solved [91]; this peptide shared the disulphide connectivity of mr3a, but surprisingly displayed a structure, described as a “flying bird motif” that is far more similar to the M1 conotoxin mr3e. It now seems apparent that not only is a conotoxin’s disulphide connectivity independent of its cysteine framework, but also that its three-dimensional structure can be independent of its disulphide connectivity [60,87,92].

Conomarphins are cysteine-free conotoxins that share a precursor sequence with the M-superfamily. The original conomarphin was identified in *C. marmoreus* [93]. Phe13 of conomarphin is modified to a D-amino acid, and was shown to be important for the peptide’s tertiary structure. Conomarphin precursors have been identified in worm-, mollusc- and fish-hunting species of *Conus* [27,94], although the activity of these peptides remains to be described.

While framework IV conotoxins of the A-superfamily were first being characterised, two conotoxins, PnIVA and B, were isolated from the venom of *Conus pennaceus* [95]. These conotoxins blocked TTX-resistant sodium channels in molluscan neurons, but had no effect on sodium currents in bovine chromaffin cells or in rat brain synaptosomes. Several years later the complete precursor sequence of PnIVB was identified [96] and indicated that, somewhat surprisingly, this peptide belonged to the M-superfamily.

VxII, isolated from the venom of *C. vexillium* [97], exhibited the unusual type II cysteine framework (previously limited to the A-superfamily conotoxin SII), although 5′ and 3′ RACE-PCR revealed an M-superfamily signal peptide sequence. Oxidative folding of the synthetic conotoxin produced a single major product that co-eluted with the native peptide. This peptide displayed sedative effects, tail-stiffening and twisted jumping on IC injection in mice, but a molecular target was not identified. A similar precursor sequence, Cp2-DD02, has since been identified in *Conus capitanus*.

Targeted cDNA sequencing of M-superfamily conotoxin sequences in *Conus vitulinus* identified two unusual conotoxin precursor sequences [94]. These sequences, vt3.1 and vt3.2, shared the signal peptide sequence with M1/M3 conotoxins but only two cysteines, separated by a single residue, were present in the predicted mature peptide region. A predicted mature peptide of vt3.1 was synthesised and formed homodimers *in vitro*, one of which displayed no bioactivity while the other produced hyperactivity in mice on IC injection. More recently it was demonstrated that this conotoxin was a modulator of BK channel function [98], preferentially inhibiting BK channels containing the β4-subunit, and in a fashion distinct from those of other peptide toxins. Vt3.1 should serve as an important tool for studying β-subunit modulation of BK channels.

In parallel with the above finding, two recently-identified sequences, one from *C. marmoreus* (Mr038) [55], the other from *C. victoriae* (M_vc3), may define a new subclass of conotoxins. These sequences share a signal peptide sequence with the M2/4/5 branches of the M-superfamily. As with vt3.1 and 2, a pair of cysteines is observed in the predicted mature peptide region, although in this case the cysteines are separated by two residues. It could be speculated that, like vt3.1, the mature peptide products of these sequences may form bioactive homodimers. Several unusual M-superfamily precursors were also recently identified in *C. flavidus* [28]. These included precursors encoding peptides with two, five or seven cysteine residues.

A conotoxin precursor identified in a venom gland library of *C. imperialis*, Im24.11, displayed a signal peptide sequence similar to that of the M-superfamily [70]. The cysteine framework of the predicted mature peptide of this precursor is not a novel framework as the name suggests, but is in fact the previously described framework IX (C-C-C-C-C-C) that is most commonly found in P-superfamily conotoxins.

Finally, two cysteine-free conotoxins, CPY-Pl1 and CPY-Fe1, were isolated recently from two species of vermivorous *Conus* [99]. These linear peptides were rich in tyrosine residues and were grouped into a conopeptide-Y, or CPY, family. The peptides were biologically active in both mice and *Caenorhabditis elegans* and electrophysiology assays showed that they were subtype-selective inhibitors of K^+^ channels. NMR spectroscopic studies showed that the peptides were unstructured in solution but did gain some helical structure in trifluoroethanol. Subsequent cDNA sequencing of the precursor sequences of these peptides revealed that they shared an M-superfamily signal peptide sequence. A summary of the diverse pharmacological activities associated with M-superfamily conotoxins is presented in Table 3.

## 20. N-Superfamily

The N-superfamily is currently limited to a few peptide sequences identified in the venom gland transcriptome of *C. marmoreus* [55]. The predicted mature peptide displays a type XV cysteine framework, although MS/MS matching data indicated that, in venom that had been reduced, the mature peptide, of at least Mr093, was present as two products, one with framework VI/VII and one with a single pair of cysteines. It should be noted that the N-superfamily appears to be closely related to the previously described V-superfamily (described below). A sequence alignment of the precursors of these two groups of conotoxins indicates a shared signal peptide sequence, as well as cysteine framework, although they differ in the number of residues between Cys6 and Cys7.

**Table 3:** Activity of selected M-superfamily conotoxins.

	Sequence	Activity	Reference
M4/5			
μ-KIIIA	**CC**N**C**SSKW**C**RDHSR**CC** *	blocks neuronal VGSCs	[100]
κ-RIIIK	LOS**CC**SLNLRL**C**OVOA**C**KRNO**CC**T *	selectively blocks the K_V_1.2 subtype of voltage-gated K^+ ^channels	[78]
ψ-PIIIE	HOO**CC**LYGK**C**RRYOG**C**SSAS**CC**QR *	inhibits neuromuscular nAChRs	[82]
M1			
mr3e	V**CC**PFGG**C**HEL**C**Y**CC**D *	No effect in mice (IC injection).	[84]
tx3a	**CC**SWDV**C**DHPS**C**T**CC**G	excitatory behaviour in mice (hyperactivity at low doses, seizures and death at higher doses) (IC injection)	[86]
LtIIIA	Dγ**CC**γOQW**C**DGA**C**D**CC**S	enhances TTX-sensitive Na^+ ^currents	[89]
M2			
mr3a	G**CC**GSFA**C**RFG**C**VO**CC**V	All M2-conotoxins tested show excitatory behaviour in mice (IC injection)	[90]
mr3b	SKQ**CC**HLAA**C**RFG**C**TO**CC**W		
tx3b	**CC**PPVA**C**NMG**C**KP**CC** *		
tx3c	**CC**RT**C**FG**C**TO**CC** *		
PnIVB	**CC**KYGWT**C**WLG**C**SP**C**G**C**	Blocks molluscan TTX-resistant Na^+^ channels	[95]
VxII	WIDPSHY**CCC**GGG**C**TDD**C**VN**C**	sedative effects, tail-stiffening and twisted jumping in mice (IC injection)	[97]
CPY-Pl1	ARFLHPFQYYTLYRYLTRFLHRYPIYYIRY	Subtype-selective inhibitors of K+ channels	[99]
CPY-Fe1	GTYLYPFSYYRLWRYFTRFLHKQPYYYVHI		

γ, carboxyglutamate; O, hydroxyproline; *, *C*-terminal amidation.

## 21. O1-Superfamily

With over 500 precursor sequences listed in the Conoserver database [11], the O1-superfamily might appear to be the most thoroughly investigated group of conotoxins. In reality, a relatively small portion of this family has undergone any form of characterisation beyond their initial identification. The majority of O1-superfamily conotoxins share a type VI/VII cysteine framework, and all of those investigated to date adopt a I-IV, II-V, III-VI disulphide connectivity arranged in an inhibitor cysteine knot (ICK) motif [101,102,103,104,105,106]. In contrast to their similarities in cysteine framework and disulphide connectivity, O1-superfamily conotoxins exhibit a relative diversity in the amino acid composition within each of their four intercysteine loops and at their *N*- and *C*-termini—a diversity that is reflected in function. The structure-activity relationships of O1-superfamily conotoxins have been reviewed recently in considerable detail [10], providing researchers with useful information on the prediction of function from O1-conotoxin primary structure; for example, precursors encoding δ-conotoxins (which block inactivation of VGSCs) have subtle but consistent differences in their propeptide and mature peptide sequences from other O1-superfamily conotoxins. So far, the O1-superfamily is comprised of δ-, μ- (VGSC blockers), κ- (voltage-gated K^+^ channel blockers) and ω-conotoxins (VGCC blockers) (Table 4). The O1-superfamily μ-conotoxin MrVIB has attracted interest as a drug lead in the development of novel analgesics [107], while κ-PVIIA has attracted attention as a cardioprotectant [108] and ω-MVIIA (ziconotide) is presently in clinical use for the treatment of chronic pain [109].

In terms of primary structure and function, Conotoxin-GS and TVIIA, isolated from the venoms of *C. geographus* [110] and *Conus tulipa* [111], respectively, appear to constitute a separate group of O1-supefamily conotoxins from those listed above. Conotoxin-GS was isolated from the venom of *C. geographus* as a fraction that produced VGSC inhibition. Sequencing revealed a 34-residue peptide with cysteine framework VI/VII. In contrast to the μ-conotoxins MrVIA and B described above, conotoxin-GS appears to bind to site I of the VGSC as evidenced by competition with [^3^H]-Lys-TTX and [^3^H]Pr-CGIIIA (an M-superfamily μ-conotoxin) binding at electroplax membranes. It should be noted that, although conotoxin-GS appears to share the binding site of M-superfamily μ-conotoxins, there is little to no structural similarity. Recently the full precursor sequence of conotoxin-GS was identified in the venom gland transcriptome of *C. geographus* [73], confirming that it is indeed a member of the O1-superfamily.

The precursor sequences of several O1-conotoxins displaying type I and XIV cysteine frameworks have been identified in the unusual *C. californicus* [112]. Additionally, of the O1-superfamily precursors identified in a recent transcriptomic study of *C. geographus* [73], several display unusual cysteine frameworks (G19, G27, G33 and G34). Interestingly, G19 had an odd number of cysteines. In a recent screen for inhibitors of rNa_V_1.7, conotoxin GVIIJ was identified by assay-directed fraction of *C. geographus* venom [113]. *N*-terminal sequencing of the peptide uncovered a partial sequence that matched the predicted mature peptide of the unusual G19 transcript. Subsequent MS/MS sequencing of the peptide revealed a bromotryptophan modification, a hydroxyproline and, for the first time in conotoxins, an S-cysteinylated-cysteine modification, accounting for the extra cysteine. Synthesis and further testing of two analogues (S-glutathionylated (GVIIJ_SSG_) and free cysteine-containing (GVIIJ_SH_) variants) showed that this peptide was an inhibitor of TTX-sensitive rNa_V_1 subtypes. Importantly the peptide forms a disulphide tether with the Na_V_1 pore loop of domain II (now termed site 8), a novel interaction and novel binding site. GVIIJ is expected to serve as an important tool for understanding the pharmacology and function of VGSCs.

Several O1-superfamily precursors encoding cysteine-free peptide products have also been identified [27,28]. Two of these, 8- and 20-residue cysteine-free peptides, were confirmed in the venom of *C. flavidus*, by MS/MS matching [28]. Both peptides were amidated and one contained a γ-carboxyglutamate modification.

**Table 4:** Activity of selected O1-superfamily conotoxins.

	Sequence	Activity	Reference
ω-MVIIA	**C**KGKGAK**C**SRLMYD**CC**TGS**C**RSGKC*	Ca_V_2.2 VGCC blocker	[114]
μ-Conotoxin-GS	A**C**SGRGSR**C**OOQ**CC**MGLR**C**GRGNPQK**C**IGAHγDV	VGSC inhibitor (site I)	[110]
δ-PVIA	EA**C**YAOGTF**C**GIKOGL**CC**SEF**C**LPGV**C**FG*	blocks inactivation of VGSCs	[115]
κ-PVIIA	**C**RIONQK**C**FQHLDD**CC**SRK**C**NRFNK**C**V*	voltage-gated K^+ ^channel blocker	[116]
μ-MrVIB	A**C**SKKWEY**C**IVPILGFVY**CC**PGLI**C**GPFV**C**V	selective blocker of Na_V_1.8 subtype of VGSC	[117]
μ-GVIIJ	GW**C**DOGAT**C**GKLRLY**CC**SGF**C**D§YTKT**C**KDKSSA	VGSC inhibitor (site 8)	[113]

O, hydroxyproline; *, *C*-terminal amidation; W, bromotryptophan; **§**, S-cysteinylated-cysteine.

## 22. O2/Contryphan-Superfamily

The majority of O2 conotoxins identified so far have 3 or 4 disulphide bonds with type VI/VII or XV frameworks, respectively (although contryphans have only a single disulphide). Several O2 conotoxins with cysteine framework VI/VII have been characterised at the protein level and some have shown activity specific to molluscs (Table 5). They have been classified as γ-conotoxins and are thought to target pacemaker channels in molluscan neurons [118,119,120], making them potentially useful tools to study the structure and function of receptors and ion channels that determine the physiology of the molluscan nervous system. The disulphide connectivity and three-dimensional structure are yet to be determined for any O2 framework VI/VII conotoxin. Several O2-superfamily precursor sequences encoding mature peptides with the type XV cysteine framework have been identified but not characterised [45].

**Table 5:** Activity of O2-superfamily (framework VI/VII) conotoxins.

	Sequence	Activity	Reference
TxVIIA	**C**GGYSTY**C**γVDSγ**CC**SDN**C**VRSY**C**TLF*	Targets pacemaker channels in molluscan neurons. No effect in rats (IC injection).	[118,121]
PnVIIA	D**C**TSWFGR**C**TVNSγ**CC**SNS**C**DQTY**C**γLYAFOS	Targets pacemaker channels in molluscan neurons (distinct, however, from TxVIIA). No effect in fish or fly larvae.	[119]
as7a	T**C**KQKGEG**C**SLDVγ**CC**SSS**C**KPGGPLFDFD**C**	Toxic effects on mollusc after IM injection. No effect in mice (IC injection).	[120]

γ, carboxyglutamate; W, bromotryptophan; O, hydroxyproline; *, *C*-terminal amidation.

The conotoxins now classified as contryphans share a very similar signal peptide sequence to O2-superfamily conotoxins. They are relatively small peptides (7–12 residues) characterised by a high degree of post-translational modification. Like many conotoxins, the first contryphans were identified initially as isolated venom components causing a characteristic “stiff-tail” syndrome in mice following IC injection [122]. There have been only a few studies reporting the molecular targets of contryphans. Contryphan-Vn, isolated from *Conus ventricosus*, was shown to be a voltage-gated and Ca^2+^-dependent K^+^ channel modulator [123]. Contryphan-M (*C. marmoreus*) produced a Ca^2+^-dependent block of L-type Ca^2+^ channels in mouse pancreatic β-cells [124]. Electrophysiological studies on rat DRG neurons demonstrated that contryphan Am975 inhibited high-voltage-activated (HVA) Ca^2+^ channels while Lo959 enhanced the magnitude of Ca^2+^ currents [125]. It is possible that the attenuation of the voltage-gated and Ca^2+^-dependent K^+^ channel currents by contryphan-Vn could be a secondary effect of voltage-activated Ca^2+^ channel blockade, thus contryphans could generally be described as Ca^2+^ channel modulators. A summary of the activities associated with O2-superfamily contryphans is presented in Table 6.

**Table 6:** Activity of O2-superfamily contryphans.

Contryphan-	Sequence	Activity	Reference
R/Tx	G**C**OwEPW**C** *	“stiff-tail” syndrome in mice (IC injection).	[122,126]
Des[Gly1]-R	**C**OwEPW**C** *	“stiff-tail” syndrome in mice (IC injection).	[122]
Bromo-R	G**C**OwEPW**C** *	“stiff-tail” syndrome in mice (IC injection).	[127]
Sm	G**C**OwQPW**C** *	“stiff-tail” syndrome in mice (IC injection).	[128]
Leu-P	G**C**VlLPW**C**	Body tremor and mucous secretion on fish (IM injection). Less activity than other contryphans in mice.	[129]
Tx	G**C**OwQPY**C** *	“stiff-tail” syndrome and paralysis of extremities in mice (IC injection).	[126]
Leu-Tx	**C**VlYPW**C** *	Causes folding and drooping of dorsal fins in fish (IM injection). Less activity than other contryphans in mice.	[126]
Vn	GD**C**PwKPW**C** *	Voltage-gated and Ca^2+^-activated K^+^ channel modulator. Mucous secretion in fish (IM injection).	[123]
M	NγSγ**C**PwHPW**C** *	Ca^2+^-dependent block of L-type Ca^2+^ channels (mouse pancreatic β-cells).	[124]
P/Am975	G**C**OwDPW**C** *	“stiff-tail” syndrome in mice (IC injection). HVA Ca^2+^ channel blocker (DRG neurons).	[125]
Lo959	G**C**PwDPW**C** *	HVA Ca^2+^ channel activator (DRG neurons).	[125]

γ, carboxyglutamate; W, bromotryptophan; O, hydroxyproline; *, *C*-terminal amidation; lowercase, d-epimerisation.

In terms of structure, contryphans are relatively well-characterised [129,130,131,132]. It was noticed very early on that d-Trp-containing contryphans exhibit two distinct peaks under reverse-phase HPLC conditions, indicating interconversion between two discrete conformations [128]. This interconversion, at least in contryphan-R, is the result of Cys2-Hyp3 *cis-trans* isomerisation [132]. Hydroxyproline (or proline) at position 1 of the disulphide loop appears to be necessary but not sufficient to produce the two conformational states. Contryphan-M is unique among these peptides in that it exhibits an extended *N*-terminus containing two γ-carboxyglutamate residues. γ-carboxyglutamate residues are known to chelate divalent metal ions, supporting metal ion-dependent structural changes important for function. Indeed, Ca^2+^ binding induces perturbations of several* N*-terminal residues and residues in the intercysteine loop [131]. This Ca^2+^-dependent change in conformation is necessary for the activity of the peptide [124].

All contryphans characterised so far share an intercysteine loop length of five residues and a characteristic primary structure. Recently, however, a contryphan precursor sequence was identified in the venom gland transcriptome of *C. victoriae* [27] that differed markedly from those described previously, in terms of both loop length and primary structure of its predicted mature peptide. It seems likely that this sequence represents a new subclass of conotoxin.

## 23. O3-Superfamily

Although the majority of known O3-superfamily conotoxins share the VI/VII cysteine spacing pattern observed in the majority of O1- and O2-superfamily conotoxins, they are distinguished by a unique signal peptide sequence [133]. The “Bromosleeper” conotoxin is currently the only O3-superfamily peptide to be successfully characterised [134]. Purified from *C. radiatus*, the 33-residue peptide induces lethargy, drowsiness and sleep in mice. Although the pharmacological target remains unknown, the symptomatology is similar but not identical to that of the conantokins—inhibitors of NMDA receptors. In addition to having bromotryptophan, the bromosleeper peptide has a number of other post-translational modifications: four γ-carboxyglutamate residues and two hydroxyproline residues.

Among the O3-superfamily precursors identified recently in the venom gland transcriptome of *C. geographus* [73], one (G27) displays an unusual, as yet undescribed, cysteine framework (C-C-CCC-C-C-C). A cysteine-free sequence, which shared the O3-superfamily signal peptide sequence, was also identified in the venom gland transcriptome of *C. victoriae* [27]. These sequences may represent new classes of conotoxin.

## 24. P-Superfamily

Conotoxin TxIXA, the prototypical P-superfamily conotoxin [135], was isolated directly from the venom of *C. textile* as a fraction that elicited “spasmodic” symptomology on IC injection in mice (Table 7). This behaviour mimicked that observed in a well-known mutant mouse (the spasmodic mouse), a phenotype caused by a deficit in glycine receptors. TxIXA did not compete with radiolabelled strychnine binding at cloned glycine receptors, indicating that either this is not the target of these peptides (perhaps a specific subtype is targeted) or the peptide binds in a non-competitive fashion. Elucidation of the full precursor sequence of the peptides revealed a unique cysteine framework (IX) and a unique signal sequence defining the P-superfamily. Very few other P-superfamily members have since been identified. One of these, gm9a, which shares a similar sequence and activity profile to TxIXA, has been characterised structurally [136]. This peptide adopted the ICK motif constrained by a I-IV, II-V, III-VI disulphide bonding pattern. This study also identified regions of the peptide that were unlikely to be involved (at least directly) in receptor binding. Although these peptides are clearly a substantial component of *Conus* venoms [27], the molecular target of the P-superfamily remains undefined.

**Table 7:** Activity of P-superfamily conotoxins.

	Sequence	Activity	Reference
TxIXA	G**C**NNS**C**QγHSD**C**γSH**C**I**C**TFRG**C**GAVN *	Hyperactivity and spasticity in mice (IC injection). No effect in fish. May target a glycine receptor.	[135]
Gm9a	S**C**NNS**C**QSHSD**C**ASH**C**I**C**TFRG**C**GAVN *	Hyperactivity and spasticity in mice (IC injection). May target a glycine receptor.	[136]

γ, carboxyglutamate; *, *C*-terminal amidation.

## 25. Q-Superfamily

The Q-superfamily was described recently from *C. flavidus* [28]. A group of conotoxin-like venom gland transcripts was identified whose signal peptide sequence did not match any previously-described superfamily. These transcripts encoded predicted mature peptides with cysteine framework XVI (C-C-CC) and, in one case, cysteine framework VI/VII. Peptide products derived from these Q-supefamily precursors were confirmed by MS/MS matching. By using RACE-PCR, Q-superfamily sequences were also identified in *C. quercinus* and *C. caracteristicus*.

Interestingly, one of the Q-superfamily precursors identified in *C. quercinus* encoded a conotoxin previously isolated from *C. quercinus* venom, qc16a [137], establishing a superfamily for this peptide. qc16a is 11 residues in length, with a type XVI cysteine framework and a ribbon-type disulphide connectivity (I-IV, II-III). In solution qc16a forms a simple β-turn motif and, when injected IC in mice, produced a depression phenotype. The molecular target of this peptide remains to be determined.

## 26. S-Superfamily

Conotoxin GVIIIA was the first S-superfamily conotoxin to be identified [138]. It was isolated from a fraction of *C. geographus* venom that inhibited serotonin-activated currents in oocytes expressing recombinant ligand-gated serotonin (5-HT_3_) receptors. Subsequent pharmacological characterisation showed that the peptide displaced the competitive agonist [^3^H]-zacopride in HEK293 cells stably expressing 5-HT_3_ receptors with an IC_50_ of 53 nM. αS-RVIIIA was identified from a fraction of crude venom from *C. radiatus* that caused audiogenic seizures in mice following IC injection [139]. This peptide showed the same cysteine pattern and signal sequence as GVIIIA but targeted a different type of ligand-gated ion channel—it had no effect at 5-HT_3_ receptors but potently inhibited neuromuscular nAChRs (Table 8) and also showed activity at several neuronal subtypes. More recently, conotoxin ca8a was isolated from the venom of *C. caracteristicus* [140] that shared the cysteine pattern observed in GVIIIA and αS-RVIIIA. The cDNA sequence of the peptide’s precursor was subsequently identified and allowed the cloning of several other S-superfamily conotoxins, ca8.2, ca8.3 (*C. californicus*), tx8.1 (*C. textile*) and ac8.1 (*Conus achatinus*). S-superfamily peptides are yet to be successfully produced by synthesis or recombinant expression, and all experiments have so far been limited by the small quantity of native peptide available.

**Table 8:** Activity of S-superfamily conotoxins.

	Sequence	Activity	Reference
GVIIIA	G**C**TRT**C**GGOK**C**TGT**C**T**C**TNSSK**C**G**C**RYNVHPSGWG**C**G**C**A**C**S *	Competitive inhibition of the 5-HT_3_ receptor.	[138]
RVIIIA	K**C**NFDK**C**KGTGVYN**C**GγS**C**S**C**γGLHS**C**R**C**TYNIGSMKSG**C**A**C**I**C**TYY	nAChR inhibition.	[139]

γ, carboxyglutamate; O, hydroxyproline; W, bromotryptophan; *, *C*-terminal amidation.

## 27. T-Superfamily

The T-superfamily is an example of a conotoxin superfamily for which many precursor sequences have been reported, probably reflecting this superfamily’s abundance in *Conus*, but remarkably little is known regarding their pharmacological properties.

The T-superfamily of conotoxins has been further subdivided into three groups based on cysteine framework. Framework V conotoxins contain two pairs of adjacent Cys residues separated by four, five or six amino acids (CC-----CC), with I–III, II–IV disulphide connectivity. Framework X conotoxins have the structure CC----C--C, with I–IV, II–III disulphide pairing. TxXIIIA is a unique T-superfamily conotoxin identified in *C. textile* [141], which is similar to the Type V framework conotoxins, but contains an extra Cys (CC----CCC). It is found in the venom as a homodimer but its disulphide bonding pattern has not been determined. Several cysteine-free precursor sequences belonging to the T-superfamily and one encoding a predicted mature peptide with a single pair of cysteines (PnMRCL-012) have also been reported [96,142].

Framework X conotoxins of the T-superfamily were first identified in *C. marmoreus* [25,143,144] and have since been confirmed in *C. victoriae* as well [27]. Several peptide sequences with obvious similarity to framework X conotoxins have been described in other species but were assigned to the A-superfamily [75,96,145]. MrIA and MrIB from *C. marmoreus* inhibit the noradrenaline transporter [25], and a synthetic variant of MrIA (Xen2174) entered clinical trials for treatment of postoperative pain. The molecular target of CMrX, also from *C. marmoreus*, is unknown, although it causes paralysis and death in mice on IC injection [144].

Of the few T-superfamily conotoxins with type V framework that have been tested, most have shown behavioural effects on fish [146], and two have shown effects on mice: TxVA causes hyperactivity on IC injection into mice [146], while SrVA causes depressed activity [147]. Furthermore, only two have a somewhat defined target: TxVA reduces presynaptic Ca^2+^ influx either by targeting a Ca^2+^ channel or a GPCR [148], while LtVD inhibits tetrodotoxin-sensitive Na^+^ currents in rat DRG neurons [149].

Recent studies suggest that at least some T-superfamily conotoxins target GPCRs. A transcript encoding a partial precursor sequence including a predicted mature peptide with framework V was reported from the venom gland transcriptome of *C. consors* [150]. The predicted mature peptide was synthesized with a I-III, II-IV disulphide connectivity and tested across a range of targets (including various subtypes of GPCRs, ion channels and neurotransmitter transporters). Activity was observed only at the somatostatin-3 GPCR in the form of radiotracer displacement. Further pharmacological characterisation indicated that this conotoxin was an antagonist selective for the somatostatin-3 receptor subtype. Several other T-superfamily conotoxins were synthesized, one of which (LiC32) also displayed inhibitory activity at the somatostatin-3 receptor. Solution structure determination of τ-CnVA revealed very few common structural features with ε-TxVA (the only T-superfamily structure previously reported [148]), despite sharing a cysteine framework and disulphide connectivity. It should be noted that the signal peptide of τ-CnVA was not actually identified and its assignment to the T-superfamily is therefore not definitive (several conotoxins of the C-superfamily also display a type V cysteine framework and reportedly target GPCRs [51]). A summary of pharmacological activities associated with T-superfamily conotoxins is presented in Table 9.

## 28. U-Superfamily

Two peptide sequences recently identified in the *C. victoriae* venom gland transcriptome [27] showed striking similarity to the “textile convulsant peptide” isolated two decades ago from the venom of *C. textile* [151]. The textile convulsant peptide, on IC injection in mice, induces symptoms characterised by “sudden jumping activity followed by convulsions, stretching of limbs and jerking behaviour”. The authors noted that this peptide was relatively unique and predicted that it belonged to a new undefined class of conotoxins. The recent identification of the signal peptide sequence of these almost identical peptides in *C. victoriae* confirmed that they are indeed members of a previously undefined conotoxin superfamily, the U-superfamily [27].

Although the pre- and propeptide sequences clearly differ from those of known conotoxin superfamilies, the U-superfamily peptides do share the cysteine spacing pattern (framework VI/VII) of members of the O1, O2 and O3 superfamilies. However, on comparing with conotoxins of these superfamilies it is apparent that there is little similarity either in the intercysteine loop composition or length [10]. For instance, loop 1 of the U-superfamily peptides is relatively short, with only two residues compared with ~six in the O1, O2 or O3-superfamily peptides. Despite its potent biological activity, the molecular target of the textile convulsant peptide was never identified.

**Table 9:** Activity of T-superfamily conotoxins.

	Sequence	Activity	Reference
TxVA	γ**CC**γDGW**CC**TAAO	Hyperactivity and spasticity in mice (IC injection). Suppression of gill display in fish. Reduced pre-synaptic Ca^2+^ influx at the *Aplysia* cholinergic synapse. May target a pre-synaptic Ca^2+^ channel or GPCR.	[146,148]
PVA	G**CC**PKQMR**CC**TL *	No effect in mice (IC injection). Suppression of gill display in fish.	[146]
AuVA	F**CC**PFIRY**CC**W	No effect in mice (IC injection). Suppression of gill display in fish.	[146]
SrVA	IINW**CC**LIFYQ**CC**	Depressed behavioural activity in mice (IC injection).	[147]
LtVD	D**CC**PAKLL**CC**NP	Inhibition of TTX-sensitive Na^+^ currents.	[149]
MrIA	NGV**CC**GYKL**C**HO**C**	Akinesia and seizures in mice (IC injection). Non-competitive inhibition of the norepinephrine transporter.	[25,143,144]
MrIB	VGV**CC**GYKL**C**HO**C**	Non-competitive inhibition of the norepinephrine transporter.	[25]
CMrX	GI**CC**GVSF**C**YO**C**	Paralysis in mice (IC injection).	[144]
τ-CnVA	E**CC**HRQLL**CC**LRFV *	somatostatin-3 receptor antagonist	[150]
LiC32	LWQNTW**CC**RDHLR**CC** *	somatostatin-3 receptor antagonist	[150]

γ, carboxyglutamate; W, bromotryptophan; T, glycosylated threonine; O, hydroxyproline; *, *C*-terminal amidation.

## 29. V-Superfamily

vi15a, isolated from the venom of the vermivorous *C. virgo*, displayed a cysteine framework XV [152]. This framework had been observed in other superfamilies although the signal peptide sequence of vi15a did not match that of any previously described superfamily, so was designated the V-superfamily. With this information, a second V-superfamily peptide sequence, vt15.1, was identified in the vermivorous *C. vitulinus.* A partial precursor sequence of a conotoxin clearly belonging to the V-superfamily (56931) was identified recently in the transcriptome of the vermivorous *C. pulicarius* [52]. A recent study of *C. flavidus* revealed high expression and diversity of V-superfamily conotoxins in this species [28]. In addition to several precursors encoding peptides with a type XV cysteine framework, several displayed the VI/VII cysteine framework. A single peptide product for one of these precursors was identified by MS/MS matching, and corresponded only to a C-terminal portion of the predicted mature peptide with four cysteines rather than the predicted eight. This observation is reminiscent of that observed for an N-superfamily conotoxin Mr093 in *C. marmoreus* (described above). To date, there is no structural or functional information on V-superfamily conotoxins.

## 30. Y-Superfamily

The “Y-superfamily” consists, at present, of a single conotoxin, ca17a. This peptide was isolated from the venom of the vermivorous *C. caracteristicus* [153]. The precursor sequence of ca17a was determined following RACE-PCR of *C. caracteristicus* venom gland cDNA, and revealed that this conotoxin did not belong to any previously described superfamily. The structure and function of this conotoxin are unknown and it is the only conotoxin so far reported to have the type XVII cysteine framework (C-C-CC-C-CC-C). More sequences need to be identified from other *Conus* species to validate this as a true gene superfamily.

## 31. Con-Ikot-Ikots

The original con-ikot-ikot was identified and characterised from the venom of *C. striatus* [154]. Uniquely among conotoxins it displayed an effect on α-amino-3-hydroxy-5-methyl-4-isoxazole propionic acid (AMPA) receptors, inhibiting channel desensitisation. The con-ikot-ikot precursor encoded a relatively large conotoxin (86 residues) with 13 cysteine residues and a unique signal sequence among conotoxins. As recently demonstrated using X-ray crystallography, con-ikot-ikot exists as a covalent homodimer with three inter-subunit disulphides [155]. The structure of each subunit can be described as a four-helix bundle. The homodimeric toxin fills a large chamber between the amino terminal- and ligand-binding domains of the AMPA receptor, interacting primarily with the latter. Con-ikot-ikot has provided mechanistic insight into receptor desensitization, and presents a new avenue for the development of therapeutics.

A recently discovered conotoxin, p21a, showed 48% homology with con-ikot-ikot [156]. p21a defined a new 10 cysteine, 5 disulphide, 7 loop framework (XXI, CC-C-C-C-CC-C-C-C) a similar cysteine arrangement to con-ikot-ikot. Unlike con-ikot-ikot however, this conotoxin appears to form a non-covalent dimer. Multiple con-ikot-ikot precursor sequences were also recently identified in the venom gland transcriptomes of *C. geographus* [73,157] and *C. victoriae* [27], three of which shared framework XXI with p21a, and two displayed the original con-ikot-ikot framework.

## 32. ConoCAPs

ConoCAP-a, a short peptide with a single disulphide, was isolated from the venom of *Conus villipenii* [158]. It displayed 78% sequence identity to the crustacean cardioactive peptide (CCAP), a multifunctional neurohormone found in invertebrates and nanomolar ligand of a GPCR in D*rosophila*. conoCAPs were found to decrease heart rate (the opposite effect to CCAP) and blood pressure in rats.

Elucidation of the conoCAP precursor revealed a long sequence encoding multiple mature peptides, an organisation distinct from the three-domain structure typically observed in other *Conus* venom peptides, but reminiscent of numerous preprohormone precursor structures, for example those of FMRF-amides and enkephalins.

## 33. Conopressins/Conophysins

Conophysin-R, isolated from the venom of *C. radiatus*, displayed a primary structure and cysteine framework reminiscent of the neurophysin peptide family [159]. In vertebrates, neurophysins are products of the oxytocin/vasopressin prohormones, and appear to be important for importing, trafficking and secretion of the hormones. No effects were observed in goldfish or mice following injection of conophysin-R, consistent with this protein playing an equivalent role to the neurophysins, rather than being directly involved in envenomation.

Conopressins-G and -S were isolated from the venoms of *C. geographus* and *C. striatus*, respectively, based on their ability to produce a “scratching effect” following IC injection in mice [160]. Sequence analysis revealed that each conotoxin was 9 residues in length, contained a single pair of cysteines, and was remarkably similar to the mammalian neurohypophysial hormone vasopressin. In humans, vasopressin acts via three subtypes of GPCR where it has both central and peripheral effects.

Conopressin-T, isolated from the venom of *C. tulipa*, is a selective antagonist of the human V_1a_ receptor [161]. Studies of conopressin-T have revealed important information on the structure-activity relationship of vasopressin peptides; all known endogenous vasopressin peptides have a Gly at position 9 and act as agonists at their respective receptors, whereas conopressin-T, which has Val in this position, is an antagonist. Replacement of Gly with Val in the agonist oxytocin, creates an antagonist.

Other conopressins have been identified in the venoms of *C. villepinii* [162] and *C. textile* [145]. With the recent discovery of the precursor sequence, conophysin-G, in the venom gland transcriptome of *C. geographus* [73], it was confirmed that conophysins and conopressins are, like vertebrate vasopressins, products of the same peptide precursor.

## 34. Conkunitzins

Conkunitzin-S1, a 60 amino acid conotoxin, was isolated from the venom of *C. striatus* [163]. Unusually for conotoxins, no post-translational modifications were evident other than an amidated *C*-terminus and two disulphide bonds. The peptide displayed high sequence similarity to the Kunitz domain peptides dendrotoxins, K^+^ channel blockers found in black mamba venom. Indeed, the NMR-based solution structure of conkunitzin-S1 showed that the characteristic Kunitz fold was present and functional studies showed that the peptide blocked voltage-activated K^+^ channels of the Shaker family. The discovery of other conkunitzin sequences was recently reported in the venom gland transcriptomes of both *C. bullatus* [164] and *C. consors* [165].

## 35. Conodipines

Conodipine-M is a 13.6 kDa component of the venom of *Conus magus* [166]. Its sequence was partially characterised and differed from most other conotoxins in that it was made up of two polypeptide chains, an α- and a β-chain. Conodipine-M displayed phospholipase-A_2_ activity and, like other PLA_2_s, required Ca^2+^ as a cofactor. Its sequence, however, shared little identity with other PLA_2_s and therefore defined a new group of enzymes.

PLA_2_s have been reported in a wide variety of animal venoms, as well as mammalian tissues and bacteria. They catalyse the hydrolysis of the ester bond at the *sn-*2 position of 1,2-diacyl-*sn*-phosphoglycerides. In addition to enzymatic activity some of these venom PLA_2_s display potent neurotoxicity.

The structure of conodipine genes was recently revealed from the venom gland transcriptome of *C. victoriae* [27]. Conodipine precursors consist of a signal peptide sequence followed by the α-chain, a propeptide sequence and finally the β-chain. Two distinct groups of conodipines were identified in *C. victoriae*, each with a unique signal peptide sequence. Thus, conodipines may constitute multiple conotoxin superfamilies. Various conodipine isoforms are reportedly present in the venom gland transcriptome of *C. consors* [165], and were also reported recently in the venom of *C. geographus* [157].

## 36. Unassigned Superfamilies—*C. miles*

A recent study on the venom gland transcriptome of *C. miles* reported three groups of precursor peptides with signal peptides that did not match any previously described superfamilies [74]. These have been temporarily annotated as “superfamilies” mi1, mi2 and mi3, which encode predicted mature peptides with cysteine frameworks XIII, undescribed (C-C-C-CCC-C-C) and VI/VII, respectively.

## 37. “Divergent” Superfamilies—*C. californicus*

*Conus californicus* is endemic to the temperate north-eastern Pacific and is considered to be genetically distant from other *Conus* species, having been recently classified into its own separate clade, Californiconus [58]. It is a generalist feeder, feeding on multiple prey types—including fish, molluscs and worms and has been observed hunting as a pack and also scavenging. Its distant genetic relationship to other *Conus* and its unusual feeding behaviour are reflected in the species venom peptides. Only half of the identified precursor sequences from *C. californicus* have been assigned to known superfamilies, and, of those that have, many display an unconventional cysteine framework. 13 “divergent” superfamilies, all from *C. californicus*, are currently listed in Conoserver [11]. The divergent-MSTLGMTLL group is the only “divergent” superfamily to have been identified in other species of *Conus* [52,167].

## 38. Conoproteins

*Conus* venoms also contain a range of higher molecular weight components. In fact, several of the earliest studies on *Conus* venoms reported the presence of high molecular weight components (possibly heterodimers) with vasoactive properties in the venoms of *C. striatus*, *C. eberneus* and *C. tessulatus* [168,169,170]. More recent studies have reported several high molecular weight components in the venoms of *C. consors* [46,165,171,172] and *C. geographus* [157]. These included a hyaluronidase (conohyal) and proteins with similarity to the pore-forming echotoxins (conoporins). While these proteins are most likely involved directly in envenomation the role of others is less clear. Some proteins may be involved in the conotoxin maturation process. The cysteine-rich secretory proteins (CRISPs) Tex31 [173] and Mr30 [174,175] are examples of *Conus* venom proteins with unclear function.

The presence of angiotensin-converting enzyme-1 (ACE-1) and endothelin converting enzyme-1 (ECE-1) metalloproteases that activate vasoconstrictive peptides was recently demonstrated in the injected venoms of the piscivorous *C. purpurascens* and *Conus ermineus* [176]. ACE was also found to be highly expressed in the venom gland of the molluscivorous *C. victoriae* [27]. More research is required to understand the roles of higher molecular weight components of *Conus* venoms.

## 39. Unclassified

A number of conotoxins have not been assigned to a particular superfamily because of a lack of information on their corresponding signal peptide sequences. While it is possible that some of the following peptides may later be grouped into known superfamilies, it seems likely that others represent the first members of as yet undescribed superfamilies.

### 39.1. Conolysins

The name conolysin refers to two related cysteine-free peptides isolated from a fraction of *Conus mustelinus* venom that caused a “moonwalker” phenotype in mice upon IC injection [177]. The authors initially hypothesised that the conotoxins were excitotoxins and tested the synthetic conolysin-Mt1 on *Xenopus* oocytes expressing K^+^ channels, only to observe that within seconds the peptide disrupted the oocyte membrane, later producing visible pores. Subsequent assays demonstrated that the peptide was a potent cytolysin. Conolysin-Mt1 and -Mt2 remain the only cytolytic conotoxins so far characterised.

### 39.2. Conophans

The conophans or γ-hydroxyconophans are a group of unusually modified conotoxins isolated from venoms of the vermivorous *Conus gladiator* and *Conus mus* [178]. They are short linear conotoxins that exhibit the unusual post-translational modification D-hydroxyvaline.

### 39.3. Conomap

Conomap-Vt is an unusual linear conotoxin isolated from the venom of *C. vitulinus* [179]. It displayed homology to the myoactive tetradecapeptide family—endogenous neuromodulators found in molluscs, annelids and insects. Conomap-Vt displayed potent excitatory activity in several molluscan tissue preparations and has a D-amino acid modification that is important for its activity.

### 39.4. Conorfamides

RF-amide neuropeptides have been found in a variety of biological systems. The isolation of Conorfamide-Sr1 and -Sr2 from the venom of *Conus spurius* [180] was the first report of this neuropeptide family in animal venom. RF-amide neuropeptides have diverse functions in invertebrate and mammalian systems, including modulation of opioid receptors, H^+^-gated ENaC channels and cardiovascular effects [181]. Conorfamide-Sr1 induced a hyperactivity syndrome in mice >16 days old (similar to that of the FMRF-amide neuropeptide). RF-amide neuropeptides in other species are encoded as multiple neuropeptides on the same precursor, but the gene structure of conorfamides in *Conus* has not been reported.

### 39.5. Bromoheptapeptide

The bromoheptapeptide, as the name implies, is 7 residues in length and contains a bromotryptophan modification as well as pyroglutamic acid at its *N*-terminus, an amidated *C*-terminus and a single disulphide bond [134]. It was isolated from the venom of *C. imperialis*. No behavioural effects were observed following peripheral or central injection of this conotoxin in mice. The bromoheptapeptide shares some similarities to the contryphan group of conotoxins, including its single disulphide bond, short length and various post-translational modifications

### 39.6. as25a

as25a, a conotoxin recently isolated from the venom of *Conus cancellatus* [182], displayed a novel cysteine framework (C-C-C-C-CC), designated as type XXV. IC injection of purified as25a in mice caused paralysis of the hind limbs and death. A variant of the peptide (as25b) containing two hydroxyproline modifications was also isolated.

### 39.7. RsXXIVA

The conotoxin RsXXIVA was recently isolated from the venom of *Conus regularis* [183]. Edman degradation of the isolated peptide revealed a novel cysteine framework (C-C-C-C-CC-CC designated as XXIV) and remarkably high sequence similarity with the ω-conotoxin MVIIA. Indeed, subsequent electrophysiological analysis showed that RsXXIVA inhibited Ca_V_2.2 channel currents, albeit with much lower affinity than MVIIA. The peptide displayed anti-nociceptive affects in both thermal acute pain and formalin chronic pain assays.

### 39.8. vil14a

vil14a is one of a group of related conotoxins isolated from the venoms of *C. villepinii*, *Conus floridanus floridensis* and *C. cancellatus*, all west Atlantic species [184,185]. These conotoxins (vil14a, flf14a-c and as14a-b) have a type XIV cysteine framework with a I-IV, II-III disulphide connectivity. The structure of vil14a, revealed by NMR spectroscopy [184], closely resembled that of the CS-α/α scorpion toxins, and is unique among conotoxins. The type XIV cysteine framework is common among conotoxins, being found in members of the A, I2, J, L, M, O1 and O2 superfamilies. vil14a, flf14a-c and as14a-b, although clearly related to one another, do not appear to be related, in terms of primary structure, to framework XIV conotoxins from other superfamilies. Determination of the full precursor sequences of these peptides, and identification in other species, may confirm that they constitute a new conotoxin gene superfamily.

## 40. Concluding Remarks

The discovery, in 2004, that SIVA, a K^+^ channel blocker with a type IV cysteine framework shared a signal peptide sequence with α-GI, an nAChR blocker with a type I cysteine framework, was the first illustration that conotoxins with different cysteine frameworks and functions could share a signal peptide and therefore belong to the same gene superfamily. This surprising structural and functional diversity in a single conotoxin gene superfamily, as this review highlights, is now known to be commonplace (Table 10). For example the M-superfamily includes both cysteine-poor and cysteine-rich conotoxins (with various cysteine frameworks) and even a homodimeric conotoxin. Furthermore, it contains conotoxins that block VGSCs, voltage-gated K^+^ channels, BK channels and nAChRs, as well as several others that display a variety of effects on the mammalian central nervous system.

Based on currently available information, it might seem that such functional diversity is limited to only a few superfamilies e.g., A, M or O1. However, this is likely to be a reflection primarily of the high level of investigation of these superfamilies, and we predict that similar diversity may be present in those groups that are yet to be examined as thoroughly. Similarly, molecular target identification seems to be limited largely to several of the more thoroughly-studied superfamilies. There are several superfamilies where biological activity in the mammalian central nervous system has been demonstrated, but the molecular target has not yet been identified. These “neglected” superfamilies may yield conotoxins with novel function, some of which might in turn serve as valuable biomedical research tools.

**Table 10:** Conotoxin gene superfamilies, associated cysteine frameworks and pharmacological families.

Gene Superfamily	Cysteine Frameworks	Pharmacological Family (Where Defined)
A	I, II, IV, VI/VII, XIV, XXII	α,κ,ρ, other
B	other	other
B2	other	
B3	XXIV	α
C	V, XV, other	α, other
D	XV, X	α
E	XXII	
F	other	
G	XIII	
H	VI/VII	
I1	VI/VII, XI	ι
I2	XI, XIV	κ
I3	VI/VII, XI	
I4	XII	
J	XIV	α, κ
K	XXIII	
L	XIV	α
M	I, II, III, IV, VI/VII, IX, XIV, XVI	α, ι, κ, μ
N	XV	
O1	I, VI/VII, IX, XII, XIV, XVI	δ, γ, κ, μ, ω
O2	VI/VII, XIV, XV	γ, other
O3	VI/VII, other	
P	IX, XIV	
Q	XVI, VI/VII	
S	VIII	α, σ
T	I, V, X, XVI	χ, μ, τ
U	VI/VII	
V	XV, VI/VII	
Y	XVII	
con-ikot-ikot	XXI, other	other
conoCAP	other	
conopressin	other	other
conkunitzin	XIV	κ
conodipine	other	other

α, nAChR inhibitors; κ, K^+^ channel blockers; ρ, adrenoceptor modulator; ι, VGSC activators; μ, VGSC blockers; δ, block VGSC inactivation; γ, molluscan pacemaker channels; ω, VGCC blockers; σ, 5-HT_3_ receptor blocker; χ, noradrenaline transporter; τ, somatostatin receptor antagonist; other, details provided in text.

Several of the superfamilies included in this review (E, F, G, H, N, Q, U, Y and the conoCAPs) should be considered putative as they have been identified in only one or two *Conus* species to date. They display many of the hallmarks that define members of well-established superfamilies, and it will be of considerable interest to see how widespread these new groups are among *Conus*, to investigate their potentially unique activity profiles, and to validate their designation as genuine conotoxin gene superfamilies.

With improvements in high-throughput cDNA-sequencing, the number of conotoxin precursor sequences reported is expected to increase dramatically. Many of these will belong to the conotoxin superfamilies described here, although some will almost certainly be members of new, as yet undescribed, superfamilies. With this in mind, Puillandre *et al.* [3] have provided some useful guidelines for the assignment of new conotoxin superfamilies. It is hoped that the present review will serve as a valuable reference for those interpreting results from conotoxin sequencing studies.
